# Microstructural and Nanoindentation Investigation on the Laser Powder Bed Fusion Stainless Steel 316L

**DOI:** 10.3390/ma16175933

**Published:** 2023-08-30

**Authors:** Abdulaziz Kurdi, Thamer Tabbakh, Animesh Kumar Basak

**Affiliations:** 1 The Center of Excellence for Advanced Materials and Manufacturing, King Abdulaziz City for Science and Technology, P.O. Box 6086, Riyadh 11442, Saudi Arabia; akurdi@kacst.edu.sa; 2 Advanced Manufacturing Technology Institute, King Abdulaziz City for Science and Technology, P.O. Box 6086, Riyadh 11442, Saudi Arabia; 3 Advanced Materials Technology Institute, King Abdulaziz City for Science and Technology, P.O. Box 6086, Riyadh 11442, Saudi Arabia; 4 Microelectronics and Semiconductors Institute, King Abdulaziz City for Science and Technology, P.O. Box 6086, Riyadh 11442, Saudi Arabia; 5 Adelaide Microscopy, The University of Adelaide, Adelaide, SA 5005, Australia

**Keywords:** additive manufacturing, laser powder bed fusion, hardness, microstructure, stainless steel 316L

## Abstract

Additive manufacturing (AM) of stainless steel is more difficult than other metallic materials, as the major alloying elements of the stainless steel are prone to oxidation during the fabrication process. In the current work, specimens of the stainless steel 316L were made by the powder laser bed fusion (P-LBF) additive manufacturing process. These specimens were investigated by electron microscopy and micro-/nano-indentation techniques to investigate the microstructural aspects and the mechanical properties, respectively. Compositionally, a similar wrought stainless steel was subjected to identical investigation, and used as a benchmark material. The microstructure of the P-LBF-processed alloy shows both equiaxed and elongated grains, which are marginally smaller (3.2–3.4 μm) than that of the wrought counterpart (3.6 μm). Withstanding such marginal gain size refinement, the increase in shear stress and hardness of the L-PBF alloy was striking. The L-PBF-processed alloy possess about 1.92–2.12 GPa of hardness, which was about 1.5 times higher than that of wrought alloy (1.30 GPa), and about 1.15 times more resistant against plastic flow of material. Similarly, L-PBF-processed alloy possess higher maximum shear stress (274.5–294.4 MPa) than that of the wrought alloy (175.9 MPa).

## 1. Introduction

Additive manufacturing (AM) can be defined as the process that builds up a structure via the ‘bottom-up’ approach. This process makes use of computer-generated 3D models, and based on slicing geometry, the final structure is formed by a layer-by-layer deposition. In that respect, this process is very different than that of the subtractive manufacturing process. In the subtractive manufacturing process, the material is being taken off from a given block of material to fabricate the desired shape/structure [1]. There are a number of variations of the AM process, and the selection of a particular process is mainly dictated by the choice of material involved. For AM of metallic materials and alloys, powder bed fusion (PBF) [2] and directed energy deposition (DED) [3] are widely used methods. In the PBF process, a thin layer of powder is consolidated by either the laser or the electron beam and termed accordingly as the laser powder bed fusion (L-PBF) [4] or electron beam melting (EBM) [5], respectively. The laser or the electron beam acts as a heat source to consolidate the powders. Both of the processes share the same principle and advantages of the other additive manufacturing techniques. However, the PBF process has a significant advantage, as it does not require a support structure, which helps to build complex structures [6,7]. There are several reasons AM technologies replace traditional methods on manufacturing metallic parts. When compared to the traditional manufacturing methods, additive manufacturing is an efficient and a rapid process that produces bespoke and near net-shaped structures at lower material costs [8] with better surface finish [9]. Owing to the benefits of 3D printing, which include the simplicity of duplicating objects, product engineering, privacy requirements, and low cost, it is most often used in applications with low manufacturing rates, limited component sizes, and complicated designs [8]. Additive manufacturing methods could rapidly replicate and print out a product, which saves money and time [10]; 3D printing technologies are successfully applied in diverse industrial sectors, such as aerospace, automotive, food, healthcare and medical, architecture, and many others [11].

Out of numerous metallic materials and alloys, stainless steel 316L (SS 316L) is a common and trusted engineering material, particularly for applications that require corrosion resistance, such as electrical industries, construction industries, food processing industries, bio-medical industries [12], and others [13]. SS316L is a common material for the above-mentioned applications, and components made from SS316L are commonly fabricated via subtractive manufacturing. However, the main advantages of the AM of SS316L components is the ease of fabrication to produce near net-shaped complex geometry orientated parts, which are economical, efficient, and material conserving compared to subtractive manufacturing [14]. Austenitic stainless steel of the 300 series has excellent mechanical properties, together with corrosion/corrosion–wear resistance [15]. The letter “L” on the material name stands for low carbon grade in the range of 0.03% [16]. Having a lower percentage of carbon contents helps towards preserving corrosion resistance, while allowing hot fabrication and welding [17]. Traditionally, this material is fabricated via melting, followed by casting and forging in the form of blocks/rods/bars, etc. Later on, these blocks are subjected to subtractive manufacturing to give the final shape of the components. Opposed to that, AM offers a one-step fabrication process of this material to the final shape of the product, and thus gains attention from scientific and engineering communities [18].

As widely reported in literature [19,20,21,22], the microstructure of the additively manufactured metallic materials is exclusive, and completely dissimilar to that of the wrought counterparts of similar compositions. The reason behind that is the high cooling rate during AM, which is a couple of magnitudes higher than that of traditional casting [23,24]. This high cooling rate induces different microstructures in the alloy and has a noticeable effect on the mechanical properties. Guan et al. [25] explored the effect of the input parameters on the strength of stainless steel 304, which was made by selective laser melting (SLM). According to their study, an increase in the powder layer thickness decreases the tensile strength when the loading is parallel to the build orientation. Layer thickness outcasts the effect of other input parameters, such as scan strategy, speed, and overlap rate. However, it was not noticed when the loading direction was perpendicular to build direction, and therefore, induces anisotropy in the mechanical properties, which are not uncommon in AM-processed materials [26]. This anisotropy arises as the highest thermal gradient, which lies in the build direction, and the grains become elongated in that direction compared to that of the perpendicular direction. In simple form, anisotropy of mechanical properties of metallic materials means that same specimen will exhibit different mechanical properties with respect to loading direction. Stainless steel specimens obtained by traditional technology show isotropic behaviour of the mechanical properties [20,21,27]. However, this became more significant in the case of L-PBF alloys, as directionality is an inherited characteristic of this technique, together with the thermal gradient that coincides with the build direction. The deep root of anisotropy in L-PBF alloys is the crystallographic texture, variation in microstructure (elongated grains in build direction), melt pool macrostructure, and associated instability and formation of defects and porosities. This was further confirmed by Liverani et al. [28], who studied how the input parameters affect the materials’ properties of SLM austenitic stainless steel 316L. According to their reports, mechanical properties were mainly affected by the build orientation, while the power of the laser and hatch space had negligible affects. There was about a 10–20% increase in yield strength and 12–13% increase in ultimate tensile strength when the laser power was increased from 100 W to 150 W, maintaining the orientation angle of 45° compared to 90°. However, the percentage elongation decreased about 50%, when the orientation angle changed from 45° to 90°. This report summarised that, while the mechanical properties were mainly affected by the building orientation, the laser power and the hatch-space played a negligible role. Tolosa et al. [29] examined the mechanical properties and their correlation with build orientations for stainless steel 316L processed by SLM. For printed stainless steel 316L with the SLM method, its mechanical properties were relatively higher compared to the wrought stainless steel 316L. From the results obtained, the yield strength of the test specimens from the different orientation were relatively higher than wrought products while maintaining high elongation values. Similar reports on the effect of the loading direction on AM fabricated specimens with respect to build orientation were also published by Li et al. [30] and Vittoria et al. [31] on the austenitic stainless steel specimens.

A critical analysis of the literature study in this topic suggests that most of the work focuses on the optimisation of input process parameters to obtain a dense component. This is usually followed by microstructural characterisation and tensile strength examination of the as-printed as well as heat-treated samples. Tensile testing of the specimens is a common technique, which offers the global tensile behaviour of the specimens. However, it takes a considerable amount of materials to prepare the ‘dog-bone’-shaped tensile specimens. Thus, the research gap exists to evaluate the mechanical properties of the P-LBF alloys through a simple but accurate way with limited usage of materials. To exploit this research gap, micro- and nano-indentation techniques were applied in the current study to obtain a similar level of mechanical properties. In spite of the simplicity, micro- and nano-indentations are powerful techniques for finding the micro- and nano-mechanical properties of a given material [19]. This is the novelty of the present work, where a simple yet powerful indentation technique was employed to assess the micro-mechanical properties of L-PBF-fabricated stainless steel 316L. In addition to that, the deformation aspects were also investigated in that length scale.

The objective of the present study is to investigate the role of the microstructure on the micro-mechanical properties of the L-PBF stainless steel 316L. In addition to that, anisotropic and deformation behaviours were also further investigated. The results of the present work expand the understanding of the AM for such alloy, together with further enhancement of the process.

## 2. Materials and Methodology

### 2.1. Materials and L-PBF of the Specimens

**Figure 1:**
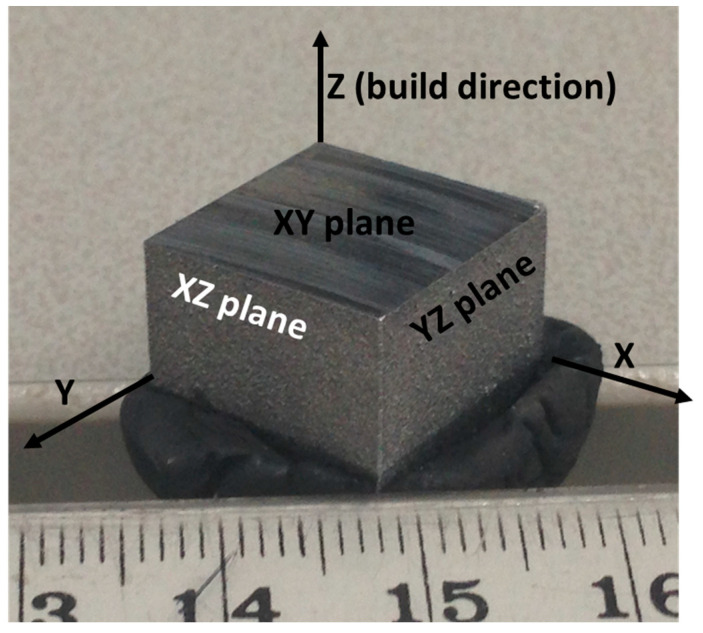
Optical image of currently investigated L-PBF fabricated stainless steel 316L.

The material investigated in the present study was the stainless steel 316L with the following composition: 16–18% Cr, 10–14% Ni, 2–3% Mo, 2% (max.) Mn, 1% (max.) Si, 0.045% (max.) C, and the balance was Fe. All the composition was in % wt. The gas-atomised powder of the stainless steel 316L of the above-mentioned composition was acquired from Valimet Inc., Stockton, CA, with the particle size distribution of 20–60 µm, and was used as a feedstock material. According to literature, the energy density (ED) of the L-PBF process is [32,33]: ED = P/(v_s_ h t), where P is laser power, v_s_ is speed, h is hatch space, and t is the thickness of the powder layer. To uphold an energy density of 62.5–104.2 J/mm^3^ [34], the following input parameters were selected: 320 W of laser power, 0.1 mm of hatch distance, 0.05 mm of layer thickness, and 600 mm/s of scan speed. These parameters strongly impact the product quality, as well as physical and mechanical properties of the fabricated alloys [35]. The AM system was a SLM 250 HL from SLM Solutions Group, Germany, and was equipped with a 400 W continuous wave Nd:YAG laser. To reduce the oxidation during the fabrication process, the closed loop system was purged with Ar. To assist with the build-up process, a base plate was employed and heated up at 200 °C. The subsequent scan direction was switched to 67° between consecutive layers, which helps to limit thermal stress build up [36]. Including this scanning strategy, all the input parameters were selected based on the information available in literature to achieve dense specimens. The final specimen was in the form of a rectangular block. The block was then subjected to 240 °C heating for stress relief, which accumulated during the L-PBF process [37]. The Archimedes principle was applied to evaluate the density of the as-built samples. Stainless steel 316L of a similar composition, however, was processed by traditional casting (and forging), which was also obtained commercially (Rolled Alloys Ltd., Singapore), and used as a reference material subjected to identical testing. To investigate the anisotropic aspects [26], if there are any, both microstructural and mechanical investigations were conducted on different planes of the rectangular block specimen and termed accordingly. The plane that is vertical to the build direction is termed as the horizontal (XY) plane, whereas the planes that are parallel to build direction are denoted as frontal (XZ) and lateral (YZ) planes. The appearance of the as-built sample together with different planes is shown in Figure 1.

### 2.2. Experimental Details

At first, the rectangular block was sliced in the middle, with the help of a diamond saw, and was subjected to hot mounting (Cito press-10, Struers, Ballerup, Denmark), grinding, and metallographic polishing, conducted with the Struers metallographic polisher. During polishing, gradually finer grades of the diamond slurry were used in the polishing cloth, and final polishing was carried out in colloidal silica. The field emission (FE) scanning electron microscope (SEM, Hitachi SU 7000) with X-ray spectrometry (EDS) and electron back-scattered diffraction (EBSD) detectors were used for microstructural examination of the samples. Both secondary and back-scattered electron modes of imaging were conducted on unetched samples. For microhardness measurements (Vickers hardness), the Clark microhardness (CM-100AT) equipment was used at 100, 300, and 500 g load, with a 5 s holding time at peak load. A total of 15 individual indentations were carried out on each plane, as shown in Figure 1. The average of the hardness values, together with the standard deviation, was stated in the manuscript. A nanoindentor (IBIS, Fischer-Cripps Lab., Perth, Australia), mounted with a Berkovich tip was used for a nanoindentation purpose. During nanoindentation, 100 mN peak load was employed with a 2 s holding time at peak load. For statistical validity, 25 individual indentations were conducted in each plane. The load–displacement curves were reported and analysed accordingly in the nanoindentation software, which provided hardness vales and Young’s modulus values, and residual and maximum indentation depth.

## 3. Results and Discussion

The theoretical density of the stainless steel 316L is 7.98 g/cc [38]. Compared to that, L-PBF fabricated stainless steel 316L attained a 97.3% density, whereas the wrought alloy attains 99.6% density, as stated in Table 1. In literature, a wide range of density values of the L-PBF-fabricated stainless steel 316L was reported, with a minimum of 88% to a maximum of 99% [39]. The reason behind that is the use of different input parameters, which consequently affect the porosity level in the specimens and influence the density of the fabricated specimens.

### 3.1. Microstructural Characterisation

#### 3.1.1. SEM Investigation

**Figure 2:**
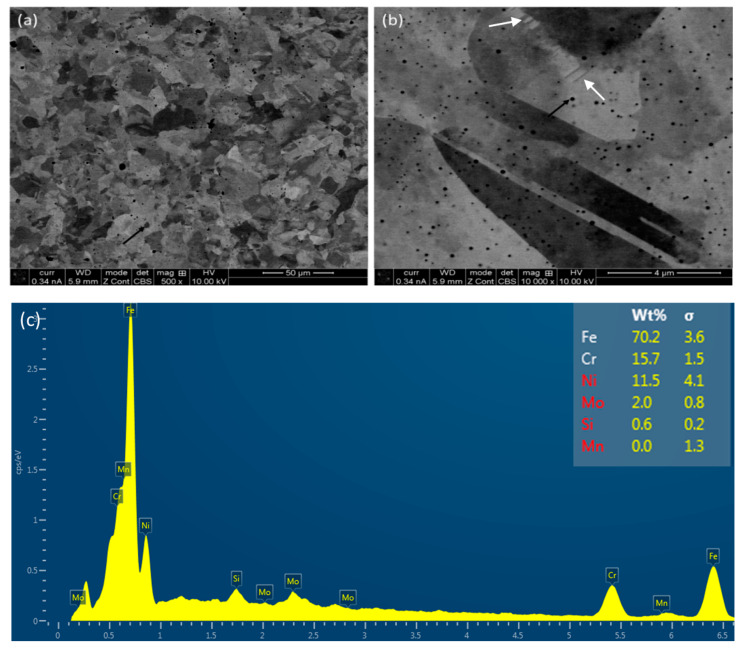
SEM micrographs on the horizontal plane of P-LBF-processed SS 316L after polishing at different magnifications (**a**,**b**), together with an elemental spectra (**c**). The black and white arrows indicate the presence of metallurgical pores and nano-twins, respectively.

Back-scattered secondary electron (BSE) micrographs on the L-PBF-processed stainless steel 316L on the horizontal plane (XY) at different magnifications are shown in Figure 2, together with elemental analysis.

**Figure 3:**
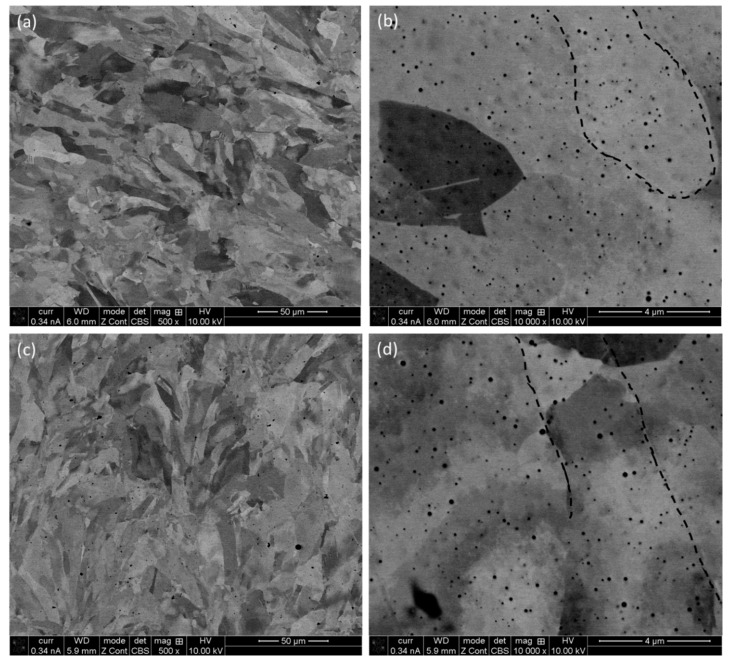
SEM images of the P-LBF-processed SS 316L after polishing on the frontal (**a**,**b**) and lateral (**c**,**d**) planes at different magnifications.

As can be seen from Figure 2a, the contrast exhibits the orientation of different grains, and the grains are equiaxed in nature. There are also numerous black spots, which are revealed as metallurgical pores at a higher magnification micrograph (Figure 2b), as shown by the black arrows (Figure 2a). These pores form due to the entrapment of gas, which cannot escape due to a high cooling rate of the fabrication process [40,41]. Some nano-twins are also evident, as marked out by white arrows [42] in Figure 2b. Figure 2c confirms the nominal composition of the printed specimens against the composition of the feedstock powder (Section 2.1). As there were no evident keyhole pores [43,44], the lack of the flow of molten metal to fill up the gaps was not an issue. Therefore, the input parameters need to be refined further to limit the pore formation, and one of the ways to achieve this is the post-heat treatment [45,46,47]. However, this was avoided in the present research, as the objective was to evaluate the role of build direction on microstructure, as well as mechanical behaviour of the as-built specimens. The micrographs obtained on the frontal and lateral planes at different magnifications are represented in Figure 3. In contrast to the grains on the horizontal plane, grains in the lateral and frontal planes (Figure 3) are elongated in nature. The reason behind that is the existence of the highest thermal gradient in that vertical (build) direction. Thus, the grains can grow along several layers, and hence give rise to elongated types of grains, as designated by the dotted lines in Figure 3b,d. The metallurgical pores are also evident throughout these planes.

As is evident from Figure 2 and Figure 3, the printed specimens suffer from porosities, which are mostly metallurgical in nature. The porosity of the specimens was not calculated in the present manuscript in a direct way. The reason behind that is that the sizes of the pores are very small (as evident in the SEM images), and therefore usual image analysis will not provide any confident results. In literature, these types of pores are generally calculated by micro-CT and other techniques, which are out of scope in the present study. However, the presence of pores and its influence was measured indirectly by density measurement. The specimen containing pores will be lighter than that of the one that does not have pores/less pores in it. Porosities are mostly influenced by the melt pool dynamics, as discussed in next section.

The melt pool dynamics are complex in nature, and have a profound effect on the formation of the microstructure, together with microstructural defeats. The profile of the melt pool was demonstrated as a long and narrow elliptical shape. The defects, such as porosity in the P-LBF alloy, occur due to the instability of the melt pool due to high energy density and irregular melt pool boundaries [48]. The oscillation and fluctuation of the melt pool surface in the horizontal and vertical direction during the cooling process directly results in defect formation, as reported by Ai et al. [49] in laser welding. As the solidification continues rapidly in the melt pool regions, the gas bubbles do not have enough time to escape the melt pool and give rise to pore formation. As the laser beam keeps moving forwards, the molten metal surface expands and oscillates because of the entrapped gas bubbles. However, the introduction of oscillation in the beam itself can facilitate the gas bubbles to escape efficiently from the melt pool, as reported by Hong et al. [50] in their numerical analysis of the melt pool behaviour [50]. This was also supported by the recent work of Fabbro et al. [51] in the case of laser welding of aluminium alloy. The top part of the melt pool is wide and shallow, whereas the bottom part is flat, which is known as the Rayleigh instability phenomena [51]. These complex melt flow characteristics are caused by the vortex, which formed in the melt pool regions. It was reported that [49] the maximum temperature and maximum flow velocity of the molten pool decreased with the increase in oscillating amplitude or frequency. This makes the melt pool become more stable and shallower, which favours the escape of the gases in bubble form. This has a positive influence in reducing porosity in the structure. As claimed by Punzel et al. [52], porosity level can be reduced to 8%, compared to 12% by the introduction of such oscillation, by introducing dual-core fiber, compared with conventional laser welding.

**Figure 4:**
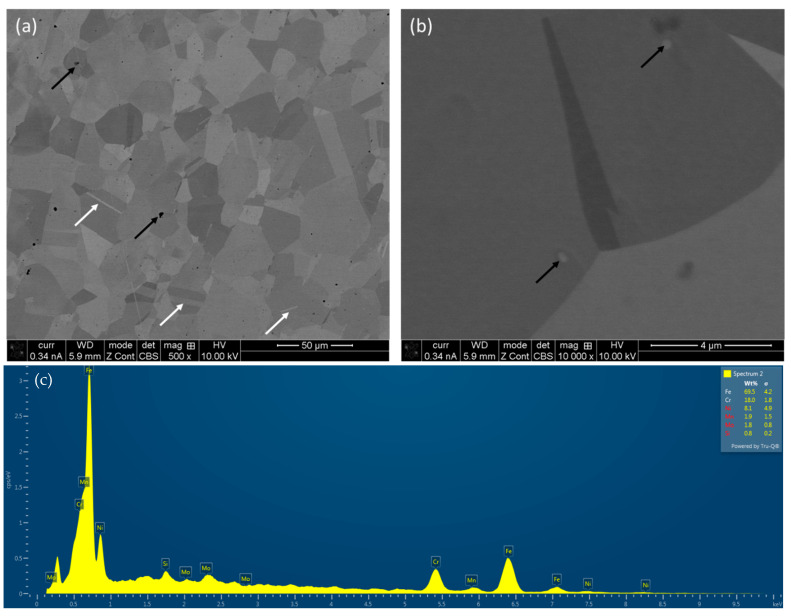
SEM micrographs of wrought SS 316L after polishing at different magnifications (**a**,**b**) together with elemental analysis (**c**).

The micrographs of the wrought alloy are shown in Figure 4 together with an elemental analysis. The major difference to that of L-PBF alloy is that there are much less metallurgical pores (as indicated by black arrows in Figure 4a), as well as the existence of twins (as indicated by white arrows in Figure 4a). During the casting process, the rate of solidification is generally much slower, together with small undercooling, which causes the development of relatively larger equiaxed grains in the presence of twins. Thus, wrought alloy is free from microstructural anisotropy. In addition, there were few precipitates, as indicated by the black arrows in Figure 4b. The elemental composition of the wrought alloy (Figure 4c) was similar to that of L-PBF-fabricated alloys. The development of grain size and texture was further analysed by EBSD, as described in the next section.

#### 3.1.2. EBSD Investigation

**Figure 5:**
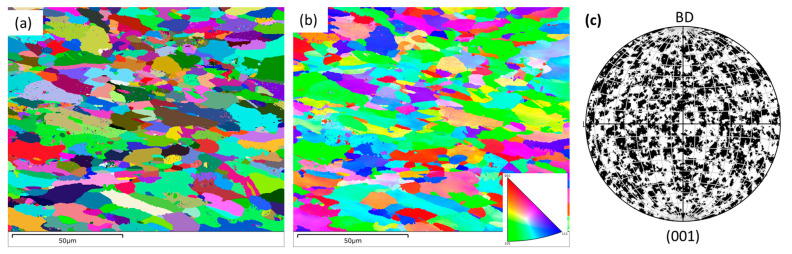
Grain boundary (GB) (**a**), inverse pole figure (IPF) (**b**), and pole figure (PF) (**c**) images on the L-PBF SS 316L alloy on horizontal planes in relation to the build direction (BD).

The results of the EBSD examination are shown in Figure 5 in the form of a grain boundary (GB) (Figure 5a), inverse pole figure (IPF) (Figure 5b), and pole figure (PF) (Figure 5c) on the horizontal plane of the L-PBF alloy. Similar to what was evident in the SEM observation (Figure 2a), the GB maps (Figure 5a) also confirm the existence of grains in different sizes and shapes in this view. In addition to that, there was no favourable direction of any particular texture development. This is mainly due to the rotation of the scan direction among subsequent layers to limit stress build up.

**Figure 6:**
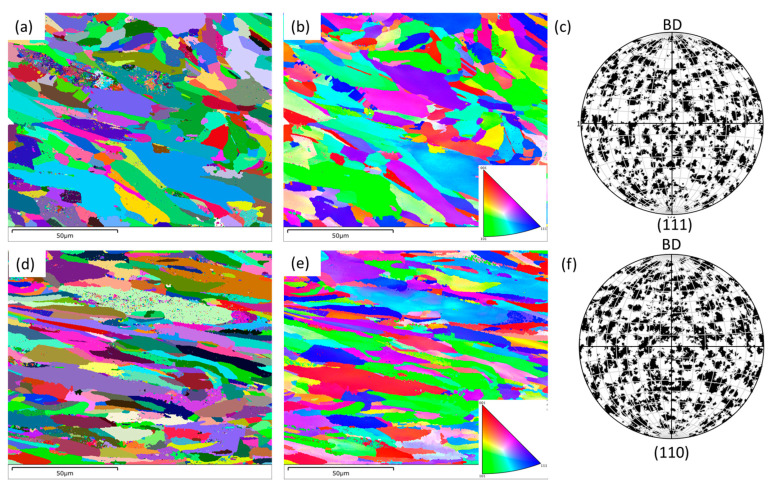
Grain boundary (GB) (**a**,**d**), inverse pole figure (IPF) (**b**,**e**), and pole figure (PF) (**c**,**f**) maps on the L-PBF SS 316L alloy on frontal (**a**,**b**) and lateral (**d**–**f**) planes in relation to the build direction (BD).

Similarly, the EBSD maps on the parallel direction to that of build orientation, i.e., on frontal and lateral directions are shown in Figure 6a–c and Figure 6d–f, respectively. From this view point, the grains seem somewhat elongated in nature. As mentioned earlier, due to the existence of thermal gradients coinciding in this direction, solidification tends to be favoured in this direction, and results in elongated grains over several layers of powder layer. Similar to that of the horizontal direction, there is also no preference in textural orientation, as confirmed by the pole figure maps (Figure 6b,e).

**Figure 7:**
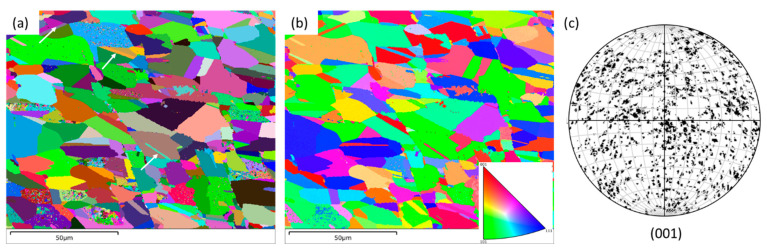
EBSD maps on wrought SS 316L alloy: (**a**) grain boundary (GB), (**b**) inverse pole figure (IPF), and (**c**) pole figure (PF) maps.

In contrast to that, ESBD figures on the wrought alloy exhibit a significant difference, as shown in Figure 7. Here, the grains are comparatively larger, with the existence of nano-twins, as marked with white arrows in Figure 7a.

**Figure 8:**
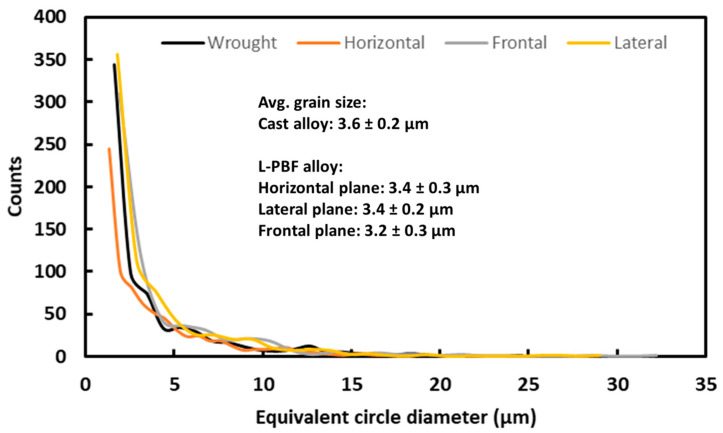
Grain size distribution together with average values of L-PBF-processed and wrought SS 316L alloy.

To have a definite conclusion on the evolution of the grain size, grain size distribution was calculated from EBSD data, as shown in Figure 8. The grain size (average values) of L-PBF alloy was about 3.2–3.4 μm, which was somewhat smaller than that of wrought alloy (3.6 ± 0.2 µm). It is worthy to note that the EBSD grain size estimates the area of a particular grain to that of an equivalent circle, and expresses the values accordingly. Therefore, though the grains in the P-LBF specimens look visually larger (Figure 2 and Figure 3), the grain size does not differ extensively. This is unique to P-LBF alloy as epitaxial grain growth taken place that can be extended among several layers. Hence, grain refinement due to the L-BPF process was not substantial compared to other metallic alloy systems, such as Al [20], Ti [21], and Ni-based super alloys [19]. This may be due to the existence of multiple major alloying elements in the SS316L that influence the melt pool dynamics differently. Further fundamental research is foreseen in this area, which is not currently available in literature and out of scope in the present work. As reported in the literature, regarding the evolution of texture in the P-LBF-processed SS316L alloy, a random texture was observed in the fusion zone [53]. The crystallographic orientation that prevailed in the P-LBF alloy has a strong role to play towards the plastic deformation of the alloy under external loading, as well as a contribution towards anisotropy in mechanical properties. During layer-by-layer formation during the P-LBF process, formation of <001> textured grains preferentially takes place along the build direction due to epitaxial grain growth. Moreover, as mentioned earlier, this direction also falls in the direction of the highest temperature gradient. According to crystallography, the <001> textured grains in this face-centred cubic (FCC) material lacks adequate slip systems [54,55], and hence contribute further towards anisotropy in the mechanical properties.

### 3.2. Mechanical Properties of the Alloys

#### 3.2.1. Vickers’s Hardness

**Figure 9:**
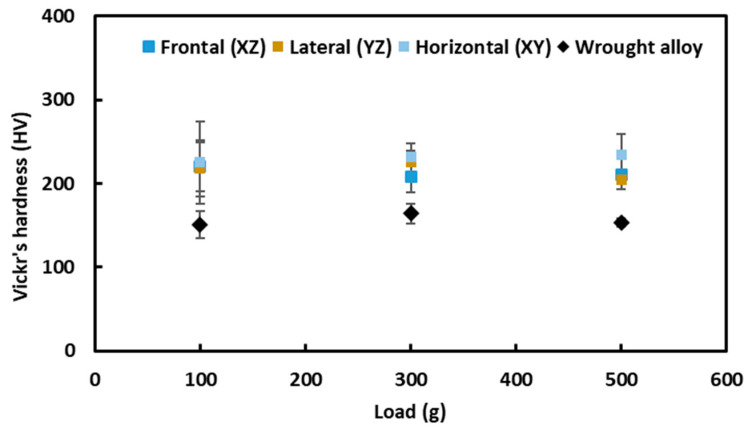
Vickers’s hardness as a function load on different planes of L-PBF-processed and wrought SS316LAl alloy.

In order to investigate the mechanical properties of the alloys, Vickers’s hardness test was carried out at different loads, namely, 100, 300, and 500 g, and the hardness values are presented in Figure 9, as well as tabulated in Table 1. The reason for choosing different indentation loads was to investigate the sensitivity of the hardness as a function of the indentation load. As is evident from Figure 8, the average Vickers’s hardness (HV_0.1_) of the L-PBF-prepared samples is in the range of 217–225 HV_0.1_, and retained similar values within the spread at different indentation loads. Though it seems the hardness on the horizontal plane is slightly higher than that of the frontal or lateral plane, the values are within the spread (error bar). Therefore, there was no profound anisotropy of hardness among different planes. In contrast to that, the hardness of the wrought alloy was about 151 HV_0.1_. Thus, the hardness of the additively manufactured alloy was about 1.5 times higher compared to the wrought alloy of a similar composition. In literature, the reported hardness varies considerably: about 178 HV [56] for wrought component and in the range of 238–302 HV [57] and 208–241 HV [58] for AM-processed alloy.

The reason for such scattered values is due to the fact that the different processing parameters of the L-PBF alloy gives rise to the variation in microstructure, together with microstructural defects, which affects the hardness values. Having said that, increasing the energy density to attain higher hardness is not the option, as increased energy density results in excessive oxidation and formation of oxide particles, which act as crack initiation sites during tensile loading of the components [59,60]. Finer grain structure in the SLM specimens showed positive improvement in micro-hardness values compared to the wrought sample, due to the Hall–Petch relation, as reported in literature [61,62].

**Figure 10:**
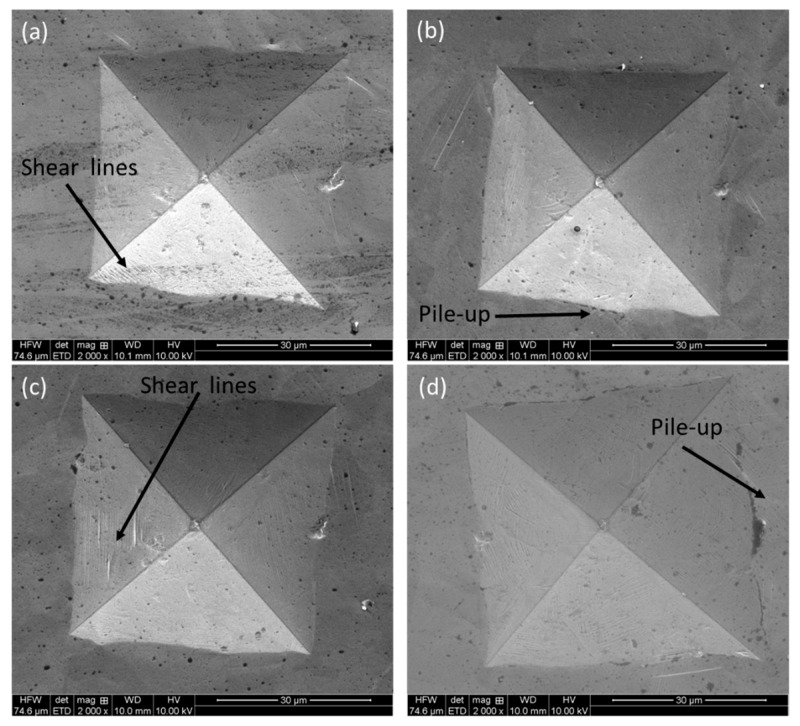
SEM micrographs on residual indentation marks after Vickers’s indentation on L-PBF SS316L alloy: (**a**) frontal (XZ), (**b**) lateral (YZ), and (**c**) horizontal (XY) along with (**d**) wrought alloy.

The SEM figures on residual indentation marks are shown in Figure 10. It is obvious that indentation cracks along the corners are absent in any case, and severe plastic deformation is evident in the form of shear lines and pile-ups, as indicated by the black arrows.

#### 3.2.2. Nano-Indentation

**Figure 11:**
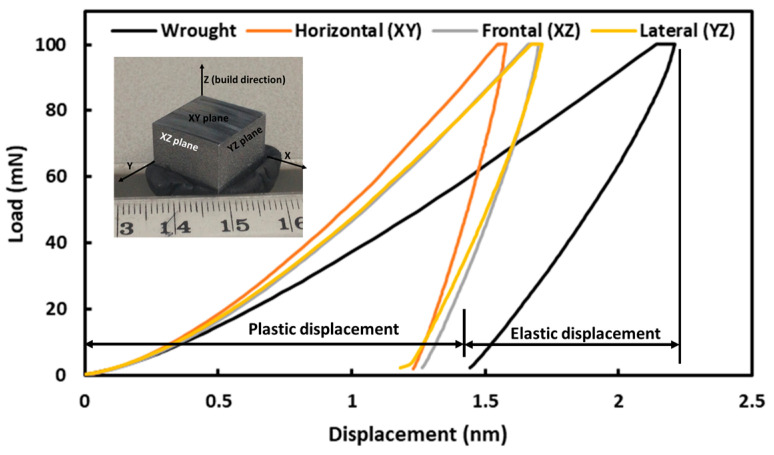
Characteristic load–displacement graphs on different planes of L-PBF-processed and wrought SS 316L.

Typical load–displacement graphs acquired in the course of nanoindentation are shown in Figure 11. Though a number of nano-indentations were carried out for the given specimens, Figure 11 contains only one load–displacement graph, recorded on each plane for better comparison. The rest of the curves are presented as supplementary (Figures S1–S4). As is evident from Figure 11, all the graphs (both on L-PBF and wrought alloy) display elastic–plastic behaviour, with predominant plasticity in nature rather than elasticity. This is more pronounced in the case of wrought alloy, which indicates relatively less hardness compared to the L-PBF-processed alloy. The portion of plastic and elastic displacement was also indicated in Figure 11, which was later used towards the calculation of resistance to plasticity.

**Figure 12:**
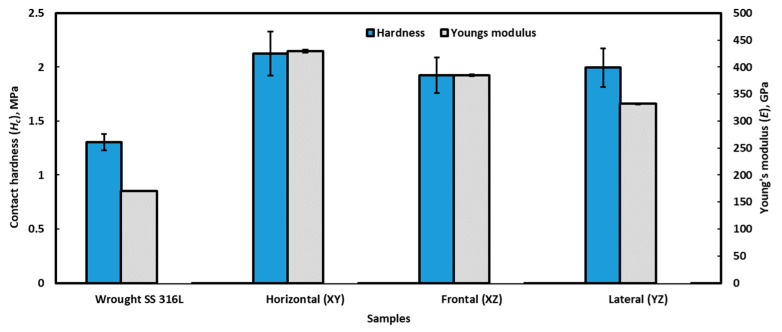
Contact hardness (*H_c_*) and Young’s moduli (*E*) of stainless steel 316L manufactured by a different method.

These load–displacement graphs were analysed (the unloading section of the graphs) to extract the contact hardness (*H_c_*) and Young’s modulus (*E*) of the specimens, as shown in Figure 12 as graphical forms and also tabulated in Table 1. These data were obtained from the nanoindentation software as output results based on the Oliver and Pharr method [63]. This method is widely accepted in literature, and equations towards that are easily accessible, and thus omitted here. Irrespective of the planes, the hardness as well as Young’s modulus is significantly higher on L-PBF-processed alloy compared to the wrought alloy. Within individual planes of L-PBF-fabricated alloy, the horizontal plane possesses a slightly higher mechanical property than that of the frontal and lateral planes. This must be related to the changes in microstructure in different directions of the L-PBF alloy, as reported in Section 3.1.1.

Furthermore, the Sakai model [64] was employed to have an in-depth understanding of the plasticity behaviour of the materials. According to the Sakai model, overall displacement in the material surface during indentation can be represented as the sum of elastic (*h_e_)* and plastic (*h_p_)* displacement, as indicated in Figure 11, and can be represented by Equation (1) [64]:

(1) $$h_{t}=h_{e}+h_{p}.\;$$

To incorporate the input parameters of nanoindentation in calculation, Equation (1) can be represented as [64,65]:

(2) $$h_{t}=\sqrt{\frac{P_{max}}{\alpha_{2}E^{\prime }}}+\sqrt{\frac{P_{max}}{\alpha_{1}H_{T}}}.$$

In Equation (2), the peak indentation load is represented by *P_max_*; α_1_, and α_2_ are constant, related to the shape of the indenter, and for the presently used Berkovich indenter, it can be considered as 24.5 and 4.4, respectively. $E^{\prime }$ is the plane strain Young’s modulus, which can be stated as:

(3) $$E^{\prime }=E/\left(1-v^{2}\right)\;$$

where *v* is Poisson’s ratio, which can be considered as 0.34 for stainless steel 316L [66], and *H_T_* represents the resistance to plasticity. The rearranging of Equation (2) gives rise to Equation (4) to represent *H_T_* [67]:

(4) $$H_{T}=\frac{H_{c}\alpha_{2}E^{\prime }}{{\left(\sqrt{\alpha_{2}E^{\prime }}-\sqrt{\alpha_{1}H_{c}}\right)}^{2}}\;.$$

**Figure 13:**
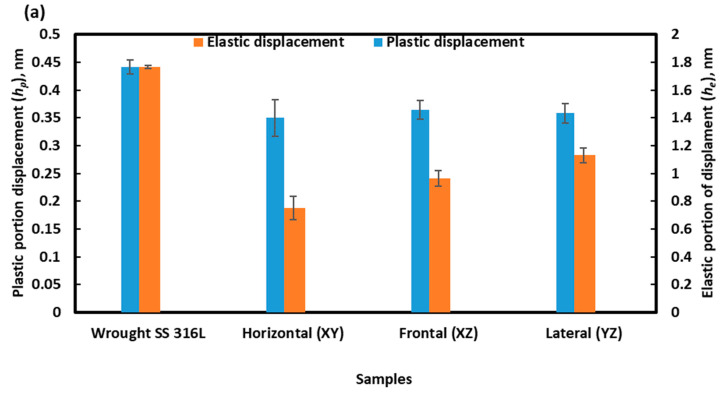

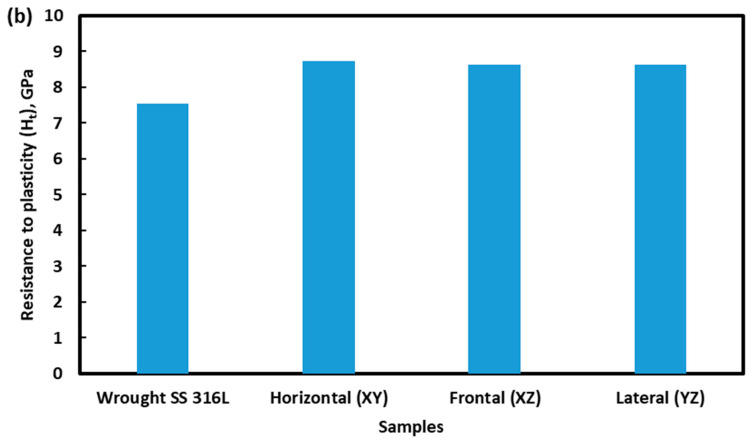
Elastic (*h_e_*) and plastic (*h_p_*) displacements (**a**) and resistance to plasticity (*H_T_*) (**b**) of the presently investigated L-PBF and cast stainless steel 316L.

Elastic (*h_e_*) and plastic (*h_p_*) portions of the displacement and contact hardness (*H_c_*) were calculated by the analysis of the load–displacement curves, whereas the resistance to plasticity was calculated accordingly to Equation (4) as graphically represented in Figure 13, and also tabulated in Table 1.

Figure 13a displays the elastic and plastic displacements induced in the sample during nanoindentation. Unsurprisingly, in the case of the wrought alloy, the extents of both elastic and plastic displacement are higher than L-PBF stainless steel 316L. Similar to hardness, the plastic displacements at different planes on the L-PBF alloy are also comparable. However, in the case of resistance to plasticity (*H_T_*) (Figure 10b), it is about 1.15 times higher for L-PBF alloy than wrought alloy. This can be attributed to minor grain refinements, induced by the L-PBF process, as explained in Section 3.1.

**Figure 14:**
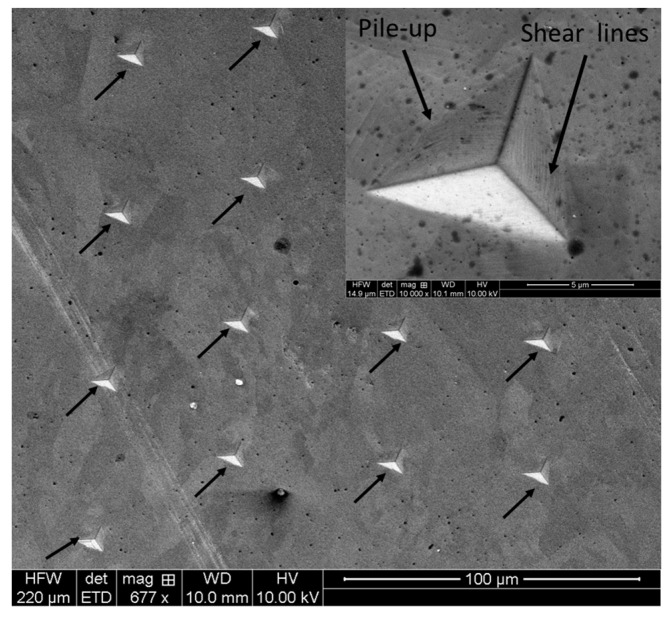
SEM image of a series of residual imprints after nanoindentation on L-PBF SS 316L alloy on the horizontal plane, along with high magnification images of one of the imprint as an insert. The arrows indicated the residual imprints.

The residual imprint of the nano-indentation marks were also looked at with a SEM, as shown in Figure 14, with high magnification images of a representative imprint, which are shown as an insert in Figure 14. Similar to what was observed in the case of micro-indentation (Vickers’s), the deformation mode is the formation of shear lines, together with material pile-up along the edges.

By incorporating the elastic modulus and indenter characteristics, the maximum shear stress experienced by the specimen in course of indentation can be calculated as follows [68]:

(5) τmax=0.45 16PcEr29π3R213 .

where the critical indentation load required to induce plasticity is *P_c_*, and the reduced elastic modulus is given by *E_r_*. The radius of the Berkovich indenter *R* was measured as 150 nm from the SEM micrograph. The critical indentation load is the point in the loading graph when the first ‘pop-in’ occurs. However, this is not evident in the present case, as the loading curves were smooth, without the presence of any ‘pop-ins’. Thus, it is required to employ the contact theory of Hertzian, where load *P* is expressed according to Equation (6) [69]:

(6) $$P=\frac{4}{3}E_{r}R^{\frac{1}{2}}h^{\frac{3}{2}}\;$$

where *h* is the instantaneous indentation depth, and the critical load *P_c_* can be shown accordingly to Equation (7):

(7) $$P_{c}=\frac{4}{3}E_{r}R^{\frac{1}{2}}{h_{e}}^{\frac{3}{2}}\;$$

It is important to note that there might be a hydrostatic core in the material just underneath the indenter during nanoindentation; however, the influence of that on the overall results will be negligible, as the material is predominantly plastic. With the help of Equations (5) and (7), maximum shear stress experienced by the samples were calculated and tabulated in Table 1. The maximum shear stress of the wrought alloy was about 175.9 MPa, and the L-PBF alloy is about 274.5–294.4 MPa. This results in about 1.5 times higher shear stress for the L-PBF-processed alloy compared to the wrought alloy. Therefore, the superior hardness and higher stress required to initiate the plastic flow of the L-PBF alloy comes down to its different microstructure to that of the wrought alloy. Moreover, the reported Young’s modulus in Table 1 is supported by the data reported in literature, where Young’s modulus of 216 ± 32 GPa for as-printed SLM and 189 ± 26 GPa for wrought SS 316 alloy was reported [70].

## 4. Conclusions

The microstructure and mechanical properties of the L-PBF-processed SS 316L was explored in the present study, and data obtained were compared against the wrought alloy. During AM fabrication, a 67° scan strategy was employed to minimise stress generation as well as anisotropy in the mechanical property. It was obvious that the microstructure of L-PBF alloy is diverse and unique, and is composed of both equiaxed and elongated grains. EBSD analysis confirms the minor grain size refinement (3.2–3.4 µm) induced by the L-PBF process. The relatively lower resistance against plastic flow of the wrought alloy was evident on the residual tracks of the Vickers’ indentation mark and was further confirmed by nano-indentation. The hardness of the L-PBF alloy (1.92–2.12 GPa) was about 1.5 times higher than that of wrought alloy (1.30 GPa). The same trend was also held for resistance of plasticity, and maximum shear stress was required to initiate the plastic flow of the material. The resistance of the plasticity of the L-PBF alloy was about 1.15 times higher than the wrought alloy, and contributed towards the higher shear stress of the L-PBF alloy (274.5–294.4 MPa) compared to 175.95 MPa for the wrought alloy.

## Figures and Tables

**Table 1:** Properties of the presently investigated stainless steel 316L made by L-PBF and casting (and forging).

Properties	P-LBF-Processed SS 316L			Wrought SS 316L (Casting and Forging)
	Lateral Plane	Horizontal Plane	Frontal Plane	
Density (gm/cc)	7.76			7.94
Hardness (HV_0.1_)	217.7 ± 33	225.1 ± 48	220.03 ± 29	151.2 ± 16
Hardness (GPa)	1.99 ± 0.17	2.12 ± 0.20	1.92 ± 0.16	1.30 ± 0.07
Young’s modulus (GPa)	331.9 ± 0.81	429.3 ± 2.47	384.8 ± 1.65	170.2 ± 0.48
Resistance to plasticity (GPa)	8.64	8.73	8.63	7.54
Maximum shear stresses (MPa)	274.5	289.8	294.4	175.95

## Data Availability

The raw/processed data used to produce the results will be made available by the corresponding author upon reasonable request.

## Supplementary Materials

The following supporting information can be downloaded at: https://www.mdpi.com/article/10.3390/ma16175933/s1, Figure S1: Typical load-displacement graphs on wrought stainless steel 316L; Figure S2: Typical load-displacement graphs on horizontal plan of L-PBF processed stainless steel 316L; Figure S3: Typical load-displacement graphs on the frontal plan of L-PBF processed stainless steel 316L; Figure S4: Typical load-displacement graphs on the lateral plan of L-PBF processed stainless steel 316L.
