# Supplementary material for: Microstructural and Nanoindentation Investigation on the Laser Powder Bed Fusion Stainless Steel 316L
Source: Materials (Basel). 2023 Aug 30;16(17):5933. doi: 10.3390/ma16175933 (PMC10488893; doi:10.3390/ma16175933)
Supplement: Supplementary file 1 [file materials-16-05933-s001.zip › materials-2526020-supplementary.pdf]

**Supplementary material:**

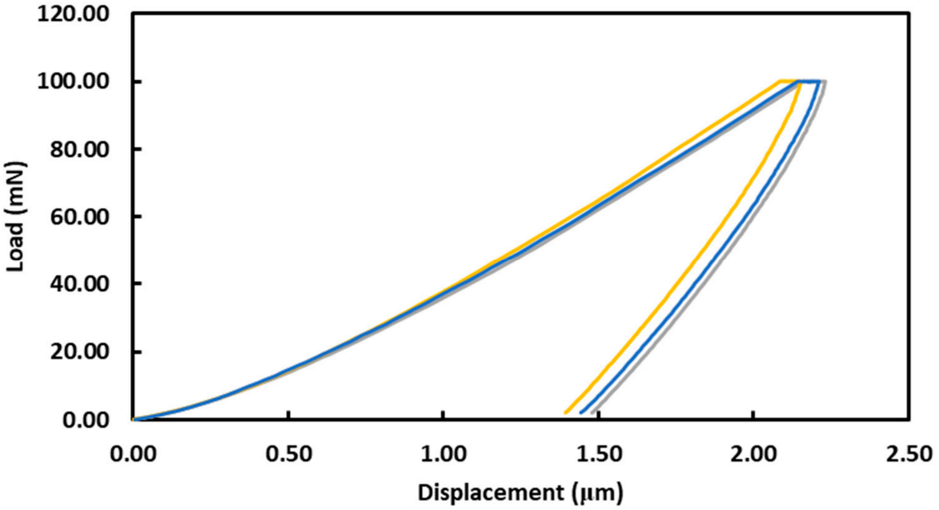


Figure S1: Typical load-displacement graphs on wrought stainless steel 316L.

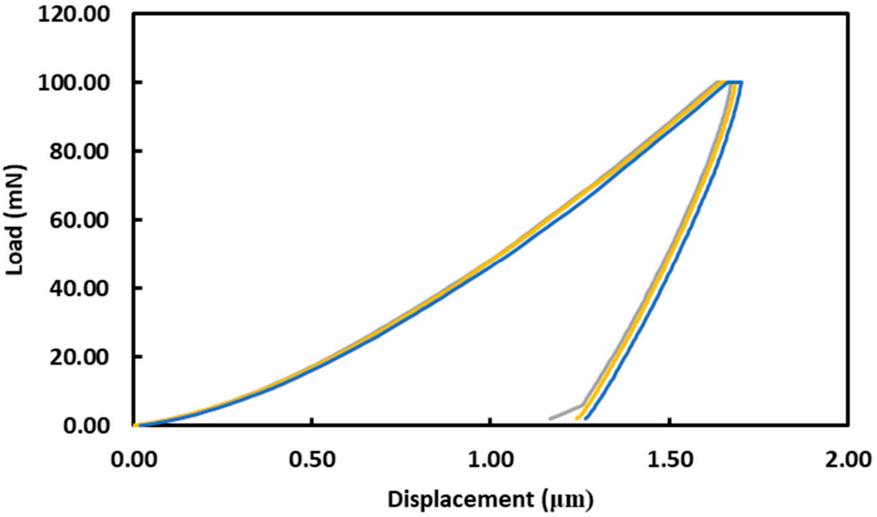


Figure S2: Typical load-displacement graphs on horizontal plan of L-PBF processed stainless steel 316L.

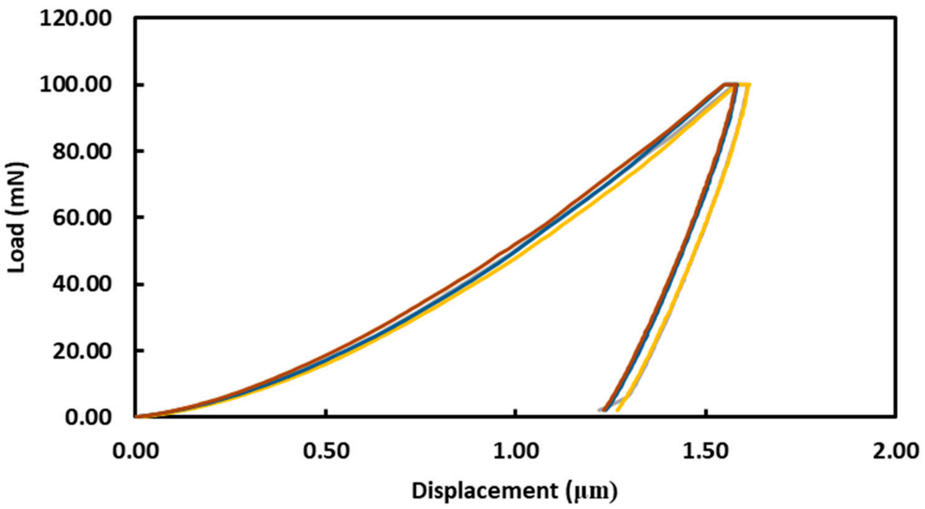


Figure S3: Typical load-displacement graphs on the frontal plan of L-PBF processed stainless steel 316L.

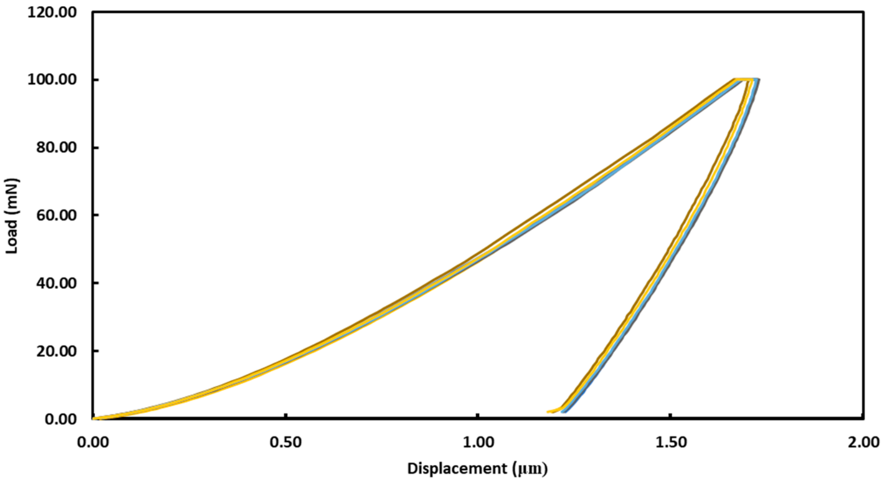


Figure S4: Typical load-displacement graphs on the lateral plan of L-PBF processed stainless steel 316L.
